# Molecular characterization and epidemiology of *Toxoplasma gondii* isolates from free-range chickens in the southwest region of Goiás: new genotypes

**DOI:** 10.1590/S1984-29612023069

**Published:** 2023-12-04

**Authors:** Natália Domann, Stéfanne Rodrigues Rezende, Amanda Cristina Corrêa Fleury, Isa Marianny Ferreira Nascimento Barbosa, Isabella da Costa Ribeiro, Júlia Batista Dornelas, Tainá Francisca Cardoso de Oliveira, Vanessa Oliveira Lopes de Moura, Heloísa Ribeiro Storchilo, Ana Maria de Castro, João Luís Garcia, Ludimila Paula Vaz Cardoso, Hanstter Hallison Alves Rezende

**Affiliations:** 1 Instituto de Ciências da Saúde, Laboratório de Bacteriologia e Micologia, Universidade Federal de Jataí – UFJ, Jataí, GO, Brasil; 2 Laboratório de Estudos da Relação Parasito-Hospedeiro, Instituto de Patologia Tropical e Saúde Pública, Universidade Federal de Goiás – UFG, Goiânia, GO, Brasil; 3 Laboratório de Protozoologia Veterinária, Universidade Estadual de Londrina – UEL, Londrina, PR, Brasil

**Keywords:** Bioassay, free-range chickens, genotyping, isolation, morphometry, Toxoplasma gondii, Bioensaio, galinhas caipiras, genotipagem, isolamento, morfometria, Toxoplasma gondii

## Abstract

The purpose of this study was to isolate *Toxoplasma gondii* from tissues of free-range chickens in the southwestern region of Goiás, to detect and molecularly characterize the genetic material of the parasite, and to determine the seroprevalence of the protozoan parasite in these animals. A seroprevalence of *T. gondii* antibodies of 76% (19/25) was found among the chickens, while genetic material from their tissues was detected in 56% (14/25). A total of 14 isolates was obtained in the bioassay, ten of which were considered acute, eight were considered isolates of high virulence lethal to mice, and four of low virulence, considered non-lethal but with the ability to chronify the infection. Seven of the ten isolates showed significant morphometric differences from the RH strain, in terms of nucleus-complex-apical distance, length and width. Genotyping of the acute isolates was performed by RFLP-PCR, using 11 genetic markers: SAG1, SAG2 (3’SAG2 and 5’SAG2), alt.SAG2, SAG3, BTUB, GRA6, c22-8, c29-2, L358, PK1, and APICO. The results were compared and classified according to the genotypes listed on the ToxoDB Platform, where different profiles were observed indicating the presence of two known genotypes (#7 and #63) and five new genotypes (NEW 3, NEW4, NEW5, NEW6, NEW 7). The results showed high seroprevalence, isolation rate, molecular detection and genotypic variations of *T. gondii* in free-range chickens in the southwestern region of Goiás.

## Resumo

O objetivo deste estudo foi isolar *Toxoplasma gondii* de tecidos de galinhas caipiras da região sudoeste de Goiás, detectar e caracterizar molecularmente o material genético do parasito e determinar a soroprevalência do protozoário nestes animais. A soropositividade para anticorpos anti- *T. gondii,* nas galinhas caipiras, foi de 76% (19/25), enquanto a detecção do material genético dos tecidos foi de 56% (14/25). Por meio do bioensaio foi obtido um total de 14 isolados, sendo 10 agudos, dos quais oito foram considerados isolados de elevada virulência, sendo letais para camundongos; e quatro de baixa virulência, com capacidade de cronificar a infecção, considerados não letais. Em sete dos dez isolados foram identificadas diferenças morfométricas significativas em relação à cepa RH, quanto à distância núcleo-complexo apical, comprimento e largura. A genotipagem dos isolados agudos por RFLP-PCR foi realizada, utilizando-se 11 marcadores genéticos SAG1, SAG2 (3´SAG2 e 5´SAG2), alt.SAG2, SAG3, BTUB, GRA6, c22-8, c29-2, L358, PK1, APICO. Os resultados foram comparados e classificados de acordo com os genótipos presentes na Plataforma ToxoDB, nos quais diferentes perfis foram observados indicando a presença de dois genótipos conhecidos (#7 e #63) e cinco genótipos novos (NEW 3, NEW4, NEW5, NEW6, NEW 7). Os resultados demonstraram elevada soroprevalência, taxa de isolamento, detecção molecular e variações genotípicas de *T. gondii* em galinhas caipiras da região sudoeste de Goiás.

## Introduction

The etiologic agent of toxoplasmosis, *Toxoplasma gondii*, is an obligate intracellular protozoan parasite. In its evolutionary cycle, its definitive host are felines, while its intermediate hosts are warm-blooded animals, also including felines and humans. Infection by *T. gondii* in humans occurs mainly through drinking water and/or food contaminated with sporulated oocysts and undercooked meat that contains viable tissue cysts. Therefore, contamination is closely linked to the lifestyle and eating habits of individuals (Castro & Dubey, 2019; Dubey, 1998a; Kim, 2018).

Among a variety of hosts, free-range chickens (*Gallus gallus domesticus*) act as sentinels of environmental contamination by *T. gondii*, since the oocysts released in feline feces are highly resistant to the environment and can be ingested by birds through direct contact as they scratch the soil in search of food (Dubey, 2010; Rodrigues et al., 2019). For this reason, chickens are often used in research in order to isolate the parasite for epidemiological and genetic studies (Alizadeh-Sarabi et al., 2020; Fernandes et al., 2016; Geuthner et al., 2019; Khan et al., 2020; Mahami-Oskouei et al., 2017). Thus, genotyping studies of *T. gondii* isolates in free-range chickens have been conducted all over the world and have contributed to the expansion of available epidemiological information on the genetic diversity of this parasite (Moré et al., 2012; Tilahun et al., 2013; Rodrigues et al., 2019; Camillo et al., 2020; Zrelli et al., 2022; Ali Awad et al., 2023)

Studies have shown a high prevalence of *T. gondii* infection in birds in Brazil’s south (Camillo et al., 2015) and southeast regions (Lopes et al., 2016), suggesting high environmental contamination. An evaluation of the genotypic characteristics of *T. gondii* in free-range chickens in the metropolitan area of Goiânia, state of Goiás by Rezende et al. (2021) revealed that 96% of tested chickens showed seropositivity for anti-*T. gondii*, and 64% of tissue samples contained the parasite’s DNA.

The population structure of *T. gondii* exhibits three predominant clonal lineages, called types I, II and III, with widespread geographical distribution. Type I isolates are classified as highly lethal in mice, while type II and III isolates show lower lethality in these animals (Howe & Sibley, 1995; Xia et al., 2017). Shwab et al. (2014), identified 156 genotypes in Central and South America, demonstrating the great population diversity of the protozoan. Despite this, in Brazil, the most common genotypes are type BrI (virulent), BrII, BrIV (intermediate virulence) and BrIII (non-virulent); this classification is based on the mortality rate in infected mice (Pena et al., 2008; Shwab et al., 2014).

One of the techniques used in the genotypic characterization of *T. gondii* is the polymerase chain reaction-restriction fragment length polymorphism (PCR-RFLP) method for ROP18 and ROP5 gene polymorphism, which detects minimal variations in a gene, in which a single base substitution can create or eliminate a site that can be digested by restriction endonuclease. (Su et al., 2006; Shwab et al., 2016; Dubey et al., 2020). The use of this technique allows for the evaluation of the diversity of this protozoan population and the identification of genetic factors that affect its virulence, thus shedding light on mechanisms of genotypic selection according to the host and the different clinical manifestations of toxoplasmosis (Dardé et al., 2007).

Knowledge about the phenotypic and genotypic characteristics of *T. gondii* isolates requires an understanding of the complex host-parasite relationship (Saraf et al., 2017). However, pathogenicity is influenced by several factors, such as host susceptibility, duration of the infection, and virulence of the isolate (Sanders et al., 2017; Alizadeh et al., 2018; Jeffers et al., 2018). There are few studies that demonstrate the *T. gondii* genotypes in circulation in Goiás. Therefore, the purpose of this study was to determine the seroprevalence of *T. gondii*, and to isolate and molecularly characterize the parasite in free-range chickens in the southwest region of the state of Goiás, Brazil.

## Material and Methods

### Characterization of the study area

**Figure 1:**
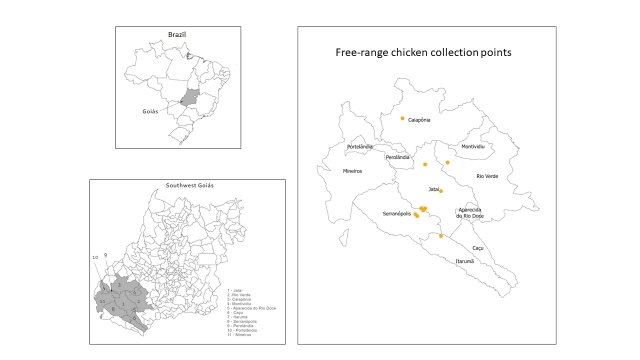
Microregion of southwestern Goiás, Brazil.

The study was conducted in four of the eighteen municipalities in southwest Goiás, which cover an area of 56,112 km^2^ (Figure 1). The economic activities carried out in this region include agriculture, agricultural production, and other services (IBGE, 2020). Data from the Mauro Borges Institute (IMB, 2018) identify the state of Goiás as the ninth largest Brazilian economy, representing 2.8% of the national GDP. This position is the result of the evolution of agribusiness, commerce, and the industrial sector.

### Chickens used in the experiment

This study involved a total of 25 adult free-range chickens, raised extensively on rural farms, which were randomly selected by their owners. The farms were selected according to the availability of donation and/or purchase of the animals, based on the criteria of being raised free and in contact with the soil, freely consuming food available in the environment, with or without supplementation with chicken feed. Among the 25 chickens used in the study, 52% (13/25) were obtained in the municipality of Serranópolis, 24% (6/25) in Jataí, 8% (2/25) in Aporé, 8% (2/25) in Caiapônia, and 8% (2/25) in Rio Verde.

The chickens were euthanized by means of cervical dislocation, followed by exsanguination. Their blood was collected in sterilized plastic bags, without anticoagulants, to obtain serum and perform serology tests. The chickens were then decapitated, and their organs, brain and heart were removed using a sterilized scalpel, and placed in sterilized plastic bags. To keep the blood and tissue samples fresh, they were placed in a thermal box, which was sent for the necessary analyses to the Laboratory of Clinical Biochemistry and Body Fluids in the Department of Biomedicine at the Federal University of Jataí – UFJ, state of Goiás, Brazil.

### Serological analysis

Serum samples from free-range chickens were analyzed using the Indirect Hemagglutination (IHA) essay, following the instructions of the commercial kit ToxoTest Hai Wiener Lab®. Samples were considered reactive when they had a titer of ≥ 32. Reactive samples were subjected to dilutions until the titer reached ≥ 1024.

### Bioassay on mice

Tissues (brain and heart) were weighed and macerated in a domestic processor along with 250 mL of 0.85% NaCl for each 50 grams of tissue, followed by peptic digestion with acid pepsin (Dubey, 1998b). The resulting homogenate was treated with 1000U of penicillin and 200mg of streptomycin. After preparation, 1 mL of the homogenate was inoculated intraperitoneally in each of a group of three 2-month-old Swiss mice (male and/or female). The remaining material was stored at –18 °C for subsequent DNA extraction.

The infected mice were monitored daily for 30 days to identify clinical signs, which included hair bristling, lethargy, and diarrhea. The animals that showed symptoms of infection were killed and subjected to intraperitoneal washing with 5 mL of 0.85% NaCl and examined under an optical microscope to identify *T. gondii*. Whenever the presence of tachyzoites was observed, slides were prepared for morphometric analysis. In addition, part of the intraperitoneal lavage was re-inoculated in another pair of mice in order to maintain the isolate.

After a period of 60 days, the mice that did not show acute signs of toxoplasmosis were euthanized in order to examine their brains for tissue cysts. The rest of the material was aliquoted and stored at –18 °C to preserve the sample. Immediately after euthanasia, blood was collected from the mice by cardiac puncture to obtain serum, which was used to search for anti-*T. gondii* antibodies through Indirect Immunofluorescence (IIF) (Camargo, 1964). Samples were incubated in sterile 0.85% NaCl and placed on slides containing wells sensitized with *T. gondii* tachyzoites. The titrations used were 1:20 and 1:40 for IgG. A result was considered negative when the observed tachyzoites were poorly defined, with red coloration and without fluorescence, and a result was regarded as positive when the tachyzoites were well defined and fluorescent, with greenish coloration (Aleixo, 2007).

### Analysis of mortality in mice

Mice mortality rate was analyzed during a period of 30 days after inoculation of chicken tissue (brain and heart) homogenate. Mortality was quantified according to the number of mice that died during the bioassay, divided by the total number of mice that were successfully infected, and lastly, multiplied by 100 to produce a percentage value. It should be noted that during the bioassay, all the mice that showed acute signs of toxoplasmosis were euthanized in order to minimize their pain and suffering. Each isolate was also classified as either lethal or non-lethal. An isolate was considered lethal when it caused the death of all infected mice in the group within 30 days after inoculation, while non-lethal isolates were those in which all or at least one mouse in the group survived for at least 30 days and produced tissue cysts (Shwab et al., 2016).

### Morphometric analysis of the isolates

Five slides were prepared from the intraperitoneal lavage positive for *T. gondii* tachyzoites. To each slide were added 25µL of intraperitoneal lavage, after which the slides were placed in a drying oven to dry them for 24 hours at 25ºC. The slides were then subjected to panoptic staining (Instant Prov®), following the manufacturer’s instructions.

The tachyzoites were photographed under 100x magnification, using a Zeiss® photomicroscope coupled to a Sony® digital camera. Thirty-nine images were saved and analyzed using the ImageJ® program, based on the evaluation of the morphological characteristics of the parasites, such as: differences in length, width at the height of the nucleus, and distance between the nucleus and the apical complex. These parameters were evaluated in the isolates obtained from the bioassay in mice and in the control strain (RH).

From each slide, 100 tachyzoites were documented and the values of each parameter were saved on a Microsoft Excel spreadsheet in order to calculate the average measurements of the isolates. Using the RStudio (Posit Team, 2023; R Core Team, 2023) application, the measurements of the isolates were compared with those of the RH strain by means of the *t-test*. Differences were considered significant when p ≤ 0,05.

### DNA extraction and detection of *Toxoplasma gondii* by PCR

DNA extractions were performed at the Institute of Tropical Pathology and Public Health of the Federal University of Goiás (IPTSP-UFG), using a PureLink® Genomic DNA kit, following the extraction protocol recommended by the manufacturer.

The PCRs were performed in a final volume of 25μL, containing 16.55 µL of Milli-Q water, 1.0 µL of MgCl2, 2.5 µL of 10x buffer (Invitrogen®), 1.25mM of each deoxynucleotide (dATP/ dTTP/ dGTP/ dCTP, Sigma®), 0.5 µL of each primer, which were Toxo-B5 (5'-TGA AGA GAG GAA ACA GGT GGT CG-3') and Toxo-B6 (5'-CCG CCT CCT TCG TCC GTC GTA-3'), 0.2 µL of Taq DNA Polymerase (Invitrogen®), and 2.5 µL of the DNA sample.

The amplification process consisted of initial denaturation at 95 °C (5 min), 35 cycles of denaturation at 95 °C (1 min each), annealing at 65 °C (1 min) and extension at 72 °C (1 min). followed by a final extension at 72 °C (10 min) (Santos et al., 1993). Peritoneal fluid, blood and tissues from mice infected with the RH and ME49 strains were used for the positive control, while the blood of a young uninfected mouse was used for the negative control.

### Molecular analysis

Ten isolates with high parasitism were subjected to DNA extraction, using a LUDWIG® mini kit for genomic DNA purification from blood (50 reactions), following the manufacturer’s instructions. The RFLP analysis involved the use of 11 genetic markers, namely, SAG1, *SAG2* (*3’SAG2* and *5’SAG2*), alt. SAG2, SAG3, BTUB, GRA6, c22-8, c29-2, L358, PK1 and APICO (Su et al., 2006; Dubey & Su, 2009). As isolates of reference, we used the type I (GT1), type II (PTG) and type III (CTG) strains and as atypical isolates we used TgCgCa1 (or Cougar), MAS, TgCatBr5, TgCatBr64 and TgRsCr1 (Su et al., 2006).

The results of agarose gel electrophoresis were analyzed and the genotypes classified using the ToxoDB platform (Kissinger et al., 2003) toxo. The identified genotypes were combined with the control genotypes, using SplitsTree version 5 software (Huson & Bryant, 2006).

## Results

### Serology and detection of *Toxoplasma gondii* DNA in tissues of free-range chickens

The presence of anti-*T. gondii* was detected by IHA in the serum of 76% (19/25) of the free-range chickens analyzed, with titers ranging from 1/32 to 1/128. Among the seropositive chickens, 8/19 came from the municipality of Serranópolis.

*T. gondii* genetic material was detected in 56% (14/25) of tissue homogenate samples from free-range chickens. However, four of the samples in which the parasite's genetic material was detected resulted in non-isolation of the parasite. Moreover, among the total, four other samples were positive for serology and isolation, but negative for the detection of genetic material by the PCR technique. In addition, 8% (2/25) of the free-range chickens presented negative results in serology, isolation and molecular detection. On the other hand, 24% (6/25) tested positive by all the techniques employed (Table 1).

**Table 1:** Serology, molecular detection and isolation by bioassay on mice performed in free-range chickens from southwestern Goiás.

**Chicken**	**Locality**	**IHA titer**	**PCR the homogenate tissue**	**Mice positive in bioassay**	**Parasitic form**	**IFI mice chronic**	**Nomenclature**
01	Serranópolis	-	+	+	Tachyzoite	NR	TgCkBrGO16
02	Serranópolis	-	+	+	Cyst	1/40	TgCkBrGO17
03	Serranópolis	-	+	+	Cyst	1/80	TgCkBrGO18
04	Rio Verde	-	-	-	-	-	-
05	Rio Verde	32	-	-	-	-	-
06	Serranópolis	-	-	-	-	-	-
07	Serranópolis	32	-	-	-	-	-
08	Serranópolis	64	+	+	Tachyzoite	NR	TgCkBrGO19
09	Serranópolis	-	+	+	Cyst	1/40	TgCkBrGO20
10	Aporé	32	+	+	Cyst	1/80	TgCkBrGO21
11	Aporé	32	-	-	-	-	-
12	Serranópolis	64	-	-	-	-	-
13	Serranópolis	64	-	-	-	-	-
14	Caiapônia	32	-	+	Tachyzoite	NR	TgCkBrGO22
15	Caiapônia	64	-	+	Tachyzoite	NR	TgCkBrGO23
16	Serranópolis	128	+	-	-	-	-
17	Serranópolis	32	+	-	-	-	-
18	Jataí	128	+	-	-	-	-
19	Jataí	128	-	+	Tachyzoite	NR	TgCkBrGo24
20	Serranópolis	32	+	+	Tachyzoite	NR	TgCkBrGo25
21	Serranópolis	32	-	+	Tachyzoite	NR	TgCkBrGo26
22	Jataí	32	+	+	Tachyzoite	NR	TgCkBrGo27
23	Jataí	64	+	+	Tachyzoite	NR	TgCkBrGo28
24	Jataí	128	+	+	Tachyzoite	NR	TgCkBrGo29
25	Jataí	128	+	-	-	NR	-

IHA = Inhibition of hemagglutination; PCR = Polymerase chain reaction; IFI = Indirect Immunofluorescence; - (Negative); + (Positive); and NR = Non-reactive.

### *Toxoplasma gondii* isolates from tissues of free-range chickens

The bioassay enabled the isolation of *T. gondii* from 56% (14/25) of the mice. Among the total number of isolates obtained, 71.4% (10/14) corresponded to acute infection, since at least one of the mice bioassayed in the group showed symptoms of acute toxoplasmosis, and 28.6% (4/14) showed chronic toxoplasmosis, since the bioassayed mice remained asymptomatic and tested positive for anti-*T. gondii* by the IIF technique.

### Mortality of bioassayed mice

After inoculation of the homogenate, 57.1% (8/14) of the isolates were considered lethal because they caused the death of all the mice in the group, which survived from nine to fifteen days after inoculation. On the other hand, 42.9% (6/14) of the isolates were considered non-lethal because all or at least one of the mice in each group survived for at least 30 days (Table 2).

**Table 2:** Survival of mice, mortality and lethality of isolates obtained from the bioassay technique.

**Isolates**	**Post-inoculation survival rate – days**	**Survival rate/no. of infected mice**	**Mortality (%)**	**Lethality**
TgCkBrGo16	>30 days	2/3	33.3	Non-lethal
TgCkBrGo17	>30 days	3/3	-	Non-lethal
TgCkBrGo18	>30 days	3/3	-	Non-lethal
TgCkBrGo19	11 days	0/3	100	Lethal
TgCkBrGo20	>30 days	3/3	-	Non-lethal
TgCkBrGo21	>30 days	3/3	-	Non-lethal
TgCkBrGo22	10 days	0/3	100	Lethal
TgCkBrGo23	9 days	0/3	100	Lethal
TgCkBrGo24	>30 days	1/3	66.6	Non-lethal
TgCkBrGo25	12 days	0/3	100	Lethal
TgCkBrGo26	12 days	0/3	100	Lethal
TgCkBrGo27	15 days	0/3	100	Lethal
TgCkBrGo28	15 days	0/3	100	Lethal
TgCkBrGo29	15 days	0/3	100	Lethal

### Morphometric analysis of the isolates

A comparison of the means of measurements of tachyzoites from the isolates obtained in this study and those of the standard RH strain indicated that tachyzoites from 70% (7/10) of the isolates differed significantly in at least one of the evaluated parameters when compared to those of the standard RH strain (Table 3).

**Table 3:** Morphometric analysis of *Toxoplasma gondii* isolates from free-range chickens in the southwest region of Goiás, compared to the standard RH strain. The values represent mean ± standard deviation of measurements.

**Isolates**	**Core-complex apical distance (µm)**	**Length (µm)**	**Width (µm)**
Cepa RH	2.29±0.76	10.42±1.13	2.12±0.31
TgCkBrGo16	2.75±0.50	9.33±2.21*	2.08±0.29
TgCkBrGo19	3.24±0.56	11.27±2.07*	2.05±0.32
TgCkBrGo22	2.92±0.42	10.53±2.12	2.13±0.37
TgCkBrGo23	2.49±0.64	11.24±1.90*	2.04±0.27
TgCkBrGo24	2.75±0.62	11.45±2.06*	2.05±0.46
TgCkBrGo25	2.57±0.64	11.41±2.38	1.76±0.36*
TgCkBrGo26	2.20±0.59	13.91±2.34	2.30±0.57*
TgCkBrGo27	**1.90±0.51** ^*^	10.52±2.71	1.76±0.49*
TgCkBrGo28	1.87±0.49	12.31±2.79	1.85±0.48
TgCkBrGo29	2.37±0.63	10.03±2.08	1.77±0.55

* p ≤ 0.05(*t*-test) comparing the isolated strains with the standard RH strain.

### Genotypic analysis of *Toxoplasma gondii* isolates

**Figure 2:**
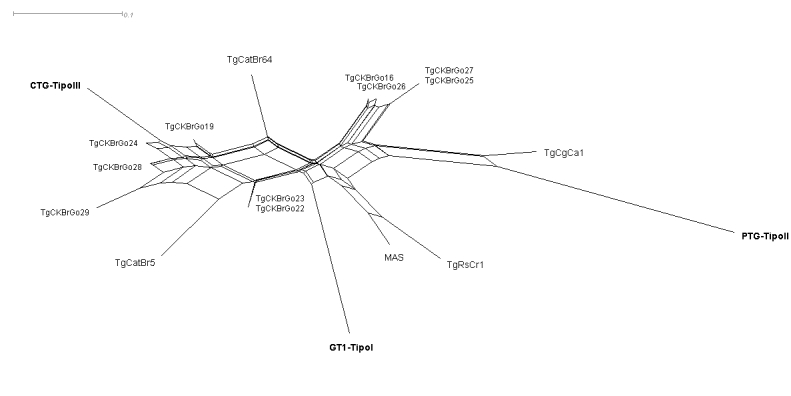
Phylogenetic tree of *Toxoplasma gondii* isolates from naturally infected free-range chickens in the southwest region of Goiás. Reference genotypes (GT1= type I, PTG = type II, CTG = type III, TgCgCa1 (or Cougar), MAS, TgCatBr5, TgCatBr64 and TgRsCr1) are included.

The genotypic characterization of the 10 isolates is described in Table 4. All the markers in 9 isolates were amplified. Among the 10 isolates, five different genotypes of *T. gondii* were identified using PCR-RFLP. Two isolates belonged to ToxoDB genotype #63 and one to genotype #7. The others have so far not been reported in the literature, the phylogenetic tree shown in Figure 2, which was created using the SplitsTree software (Huson, 1998; Huson & Bryant, 2006), indicates the diversity of isolates and the distance between the clonal archetypes of *T. gondii* in the microregion of southwest Goiás.

**Table 4:** Genotypic characterization of *Toxoplasma gondii* isolates obtained from naturally infected free-range chickens in the southwest region of the state of Goiás.

**Isolates**	**Parasitic form**	**MARKER**											
		**SAG1**	**5-3 SAG2**	**alt.SAG2**	**SAG3**	**BTUB**	**GRA6**	**c22-8**	**c29-2**	**L358**	**PK1**	**Apico**	**Genotype ToxoDB**
**TgCkBrGO24**	Tachyzoite	I	III	III	III	III	III	III	III	III	III	I	#7
**TgCkBrGO16**	Tachyzoite	I	I	II	III	III	III	I	III	I	II	I	#63
**TgCkBrGO26**	Tachyzoite	I	I	II	III	III	III	I	III	I	II	I	
**TgCkBrGO19**	Tachyzoite	I	I or III	III	III	I or III	III	I	III	III	III	I	NEW3
**TgCkBrGO22**	Tachyzoite	I	I or III	III	III	I or III	III	I	III	I	III	I	NEW4
**TgCkBrGO23**	Tachyzoite	I	I or III	III	III	I or III	III	I	III	I	III	I	
**TgCkBrGO25**	Tachyzoite	I	I or III	II	III	I or III	I	I	III	I	II	I	NEW5
**TgCkBrGO27**	Tachyzoite	I	I or III	II	III	I or III	I	I	III	I	II	I	
**TgCkBrGO28**	Tachyzoite	I	I or III	III	III	I or III	III	III	I	III	III	I	NEW6
**TgCkBrGO29**	Tachyzoite	I	I or III	III	III	I or III	I	III	I	III	*UD	I	NEW7

* UD = Undefined.

## Discussion

Studies conducted in Brazil also used the IHA technique to detect anti-*T. gondii* antibodies. Beltrame et al. (2012), who evaluated free-range chickens from eight municipalities in the state of Espírito Santo, found a seropositivity rate of 40.4%, and isolated antibodies in 75% of seropositive chickens. Ferreira et al. (2018) performed serological screening in free-range chickens in Espírito Santo, reporting a 38% seropositivity rate and obtaining 10 isolates. In turn, Rezende et al. (2021) evaluated the epidemiology of toxoplasmosis in free-range chickens in the metropolitan area of Goiânia, state of Goiás, finding a 96% positivity rate for anti-*T. gondii* and obtaining 15 isolates from the bioassay. These data indicate that, although the seropositivity rate for anti-*T. gondii* found in this study and by the other aforementioned authors is variable, they are consistent with the data reported in the literature.

The non-isolation of *T. gondii* in seropositive chickens determined here is admissible, since seropositivity for anti-*T. gondii* antibodies demonstrates that exposure to the parasite occurred, but that this exposure does not necessarily characterize its viability (Alizadeh-Sarabi et al., 2020). This finding may also be related to tissues that contain non-viable cysts, or that have a low parasite load, in addition to the fact that the viability of *T. gondii* may decline during the process of tissue digestion.

Zrelli et al. (2022), used molecular techniques to detect *T. gondii* DNA in free-range chicken viscera and meat. As a result, it was observed that the heart had a higher prevalence of infections, corroborating other studies (Dubey, 2010; Schares et al., 2018; Minutti at al., 2021). In Brazil, the consumption of raw or undercooked meat is very common, with the data observed in this study and in the literature, the consumption of chicken viscera, mainly the heart, increases the risk of contracting strains that are highly pathogenic for humans.

In this study, the isolation of *T. gondii* from seronegative chickens also occurred. The same finding has been reported by other authors, and can be attributed to recent infections, in which anti-*T. gondii* antibodies have not yet been produced, or their low titers were undetectable by the serological tests employed (Camillo et al., 2018; Dubey, 2010). Conversely, in the chronic phase, the hypothesis points to a drastic reduction in antibody titers, preventing their detection (Casartelli-Alves et al., 2014).

Upon evaluating the results of the isolation of the parasite detected in the genetic material, it was found that seropositive animals whose bioassay led to the isolation of the parasite did not show positivity in the PCR technique. In other words, *T. gondii* genetic material was not detected in these samples. This finding is explained by Camilo (2017), who used the B1 gene to amplify the genetic material of *T. gondii* and reported that successful amplification required the presence of a minimum of 100 tachyzoites in a sample. Hence, it is believed that, in this study, samples testing negative in the detection of genetic material but positive in the bioassay may have contained a parasite load below the limit of detection. Moreover, it is also possible that the genetic material of the samples became degraded, preventing DNA amplification. In addition, it should be kept in mind that studies on the isolation of the parasite, in terms of the detection of its genetic material, can be affected by the origin of the chickens, the type and quantity of tissues used, and even the type of PCR technique employed (Dubey et al., 2020).

Feitosa et al. (2016), who investigated the seroprevalence and isolation of *T. gondii* in free-range chickens in the state of Paraíba, northeastern Brazil, reported that 48.5% of the isolates were lethal to all the infected mice. The finding of those authors is consistent with those of this study, in which a high lethality rate of *T. gondii* isolates was also observed when these isolated were bioassayed in mice.

As for the biological characterization of the isolates, Rezende (2018) made a morphometric analysis of seven isolates from free-range chickens in the metropolitan region of Goiânia and found that all the isolates evaluated differed from the standard strain, suggesting that strains of the same genotype may differ in their phenotypic and virulence characteristics.

Genotyping of the 10 acute isolates resulted in the identification of five different *T. gondii* genotypes and two previously known genotypes. Two isolates belonged to genotype #63 and one belonged to ToxoDB genotype #7. The others were not previously described in the literature and were therefore considered new. None of the isolates characterized here showed a well-defined or classic clonal type I, II or III, and were therefore all identified as atypical.

In Brazil, ToxoDB genotype #7 has been identified in various locations, including the states of Maranhão (Sousa et al., 2016), Mato Grosso do Sul – Pantanal, Pará (Shwab et al., 2014) and Ceará (Oliveira et al., 2009). This reinforces the fact that genotype #7 is more common in chickens, as is the presence of many unique genotypes. Our study offers the first report of this genotype in the southwest region of the state of Goiás.

Similar to our report, Casartelli-Alves et al. (2021) provided the first description of ToxoDB genotype #63 in free-range chickens in the state of Rio de Janeiro. Although only two isolates were characterized as #63, this demonstrates that, like in other Brazilian states, high genetic variability, which is still little known, also occurs in the southwest region of Goiás. This is demonstrated in the phylogenetic tree created from the data obtained (Figure 2), which indicates the diversity of isolates and the distance between the clonal archetypes of *T. gondii* in the southwest region of Goiás. These data emphasize the high genetic variability found in Brazil.

## Conclusions

The high prevalence of anti-*T. gondii* IgG antibodies found in free-range chickens, as well as the isolation of acute and chronic strains and the detection of the parasite's genetic material, confirm the existence of *T. gondii* in the areas covered by this study, and point to the possible occurrence of contamination of animals used for human consumption. This finding justifies the inference of a potential risk of contamination to both animals and humans. Moreover, based on the morphometric analysis of the isolates identified in this study, at least one of the parameters evaluated in the tachyzoites of seven acute isolates showed a significant difference, which may be attributed to the presence of five genotypes not yet described in the literature, or already known but having characteristics that differ from those previously documented. This was verified through genotyping, which revealed different profiles in the southwest region of Goiás, five new and two known *Toxoplasma gondii* genotypes (#7 and #63).
